# Median mandibular flexure—the unique physiological phenomenon of the mandible and its clinical significance in implant restoration

**DOI:** 10.3389/fbioe.2023.1238181

**Published:** 2023-09-07

**Authors:** Jing Gao, Lulu Jiang, Baohong Zhao

**Affiliations:** Center of Implantology School and Hospital of Stomatology, China Medical University, Liaoning Province Key Laboratory of Oral Diseases, Shenyang, China

**Keywords:** median mandibular flexure, implant restoration, deformation, factors, clinical significance

## Abstract

Mandibular flexure, characterized by unique biomechanical behaviors such as elastic bending and torsion under functional loading, has emerged as a crucial factor in oral clinical diagnosis and treatment. This paper presents a comprehensive review of the current research status on mandibular flexure, drawing insights from relevant studies retrieved from the PubMed database (www.ncbi.nlm.nih.gov/pubmed), including research conclusions, literature reviews, case reports, and authoritative reference books. This paper thoroughly explores the physiological mechanisms underlying mandibular flexure, discussing different concurrent deformation types and the essential factors influencing this process. Moreover, it explores the profound implications of mandibular flexure on clinical aspects such as bone absorption around dental implants, the precision of prosthesis fabrication, and the selection and design of superstructure materials. Based on the empirical findings, this review provides crucial clinical recommendations. Specifically, it is recommended to exert precise control over the patients mouth opening during impression-taking. Those with a high elastic modulus or bone-tissue-like properties should be prioritized when selecting superstructure materials. Moreover, this review underscores the significance of customizing framework design to accommodate individual variations in facial morphology and occlusal habits. Future research endeavors in this field have the potential to advance clinical diagnosis and treatment approaches, providing opportunities for improvement.

## 1 Introduction

Weinmann and Sicher. (1955) demonstrated for the first time in 1955 that the cohesion of the traction condyle can cause the mandible to bend and deform due to the contraction of the lateral pterygoid muscle. In 1973, (Goodkind and Heringlake, 1973), designated this bending phenomenon as mandibular flexure (MF). In recent years, researchers interest in the physiological deformation of the mandible has gradually grown.

**FIGURE 1:**
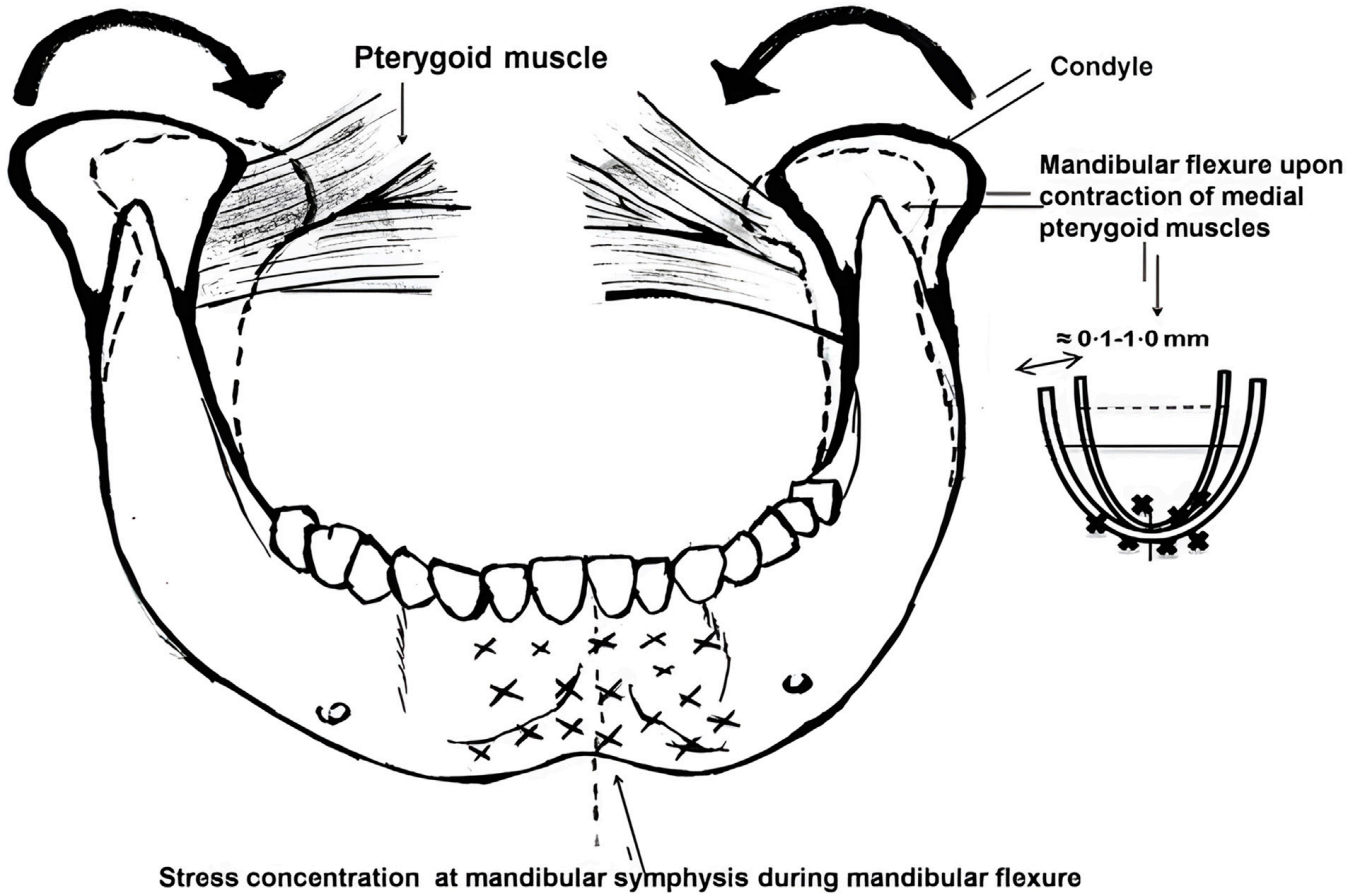
Medial rotation of the mandible and decrease in arch width during mandibular flexure caused by contraction of lateral pterygoid muscle (Sivaraman et al., 2016).

MF is a unique and complex physiological phenomenon of the mandible involving the interaction of numerous head and neck muscles (Sivaraman et al., 2016; Azpiazu-Flores et al., 2022). Generally, researchers (Regli and Kelly, 1967; Hylander, 1984) concur that the primary factor attributed to MF is the contraction of the pterygoid muscle; the platysma, mylohyoid, and superior constrictor also play a role in condyle convergence (Hylander, 1984). As the muscle attached to the mandible contracts, the tension it exerts on the mandible changes both the mandibles morphology and the teeth relative positioning. Hylanders experimental findings regarding the biological behavior of the mandible in adult rhesus monkeys reveal four jaw deformation types in MF (Hylander, 1984; Sivaraman et al., 2016) (As shown in Figure 1.).

1. Symphyseal bending associated with median convergence, or corporal approximation: this type of strain is associated with the contraction of the lateral pterygoid muscle during jaw opening movements.
2. Dorsoventral shear: this produces a shearing force in the sagittal plane and is a result of the vertical components of muscle forces from the lateral pterygoid muscles and their action forces at the condyles. The magnitude of the shear force depends on the points of application. The amount of shear force is equal on both sides of the mandible during symmetrical loading, while the amount of deformation differs between the working and balancing sides during unilateral loading.
3. Corporal rotation: this occurs during rotation of the body of the mandible, usually during the lower stroke of mastication. The resultant force causes narrowing of the dental arch.
4. Anteroposterior shear: this is induced by the contraction of the lateral components of the jaw-elevating muscles. It appears late in the power stroke, and the bending moment increases from the posterior to the anterior region.”

**FIGURE 2:**
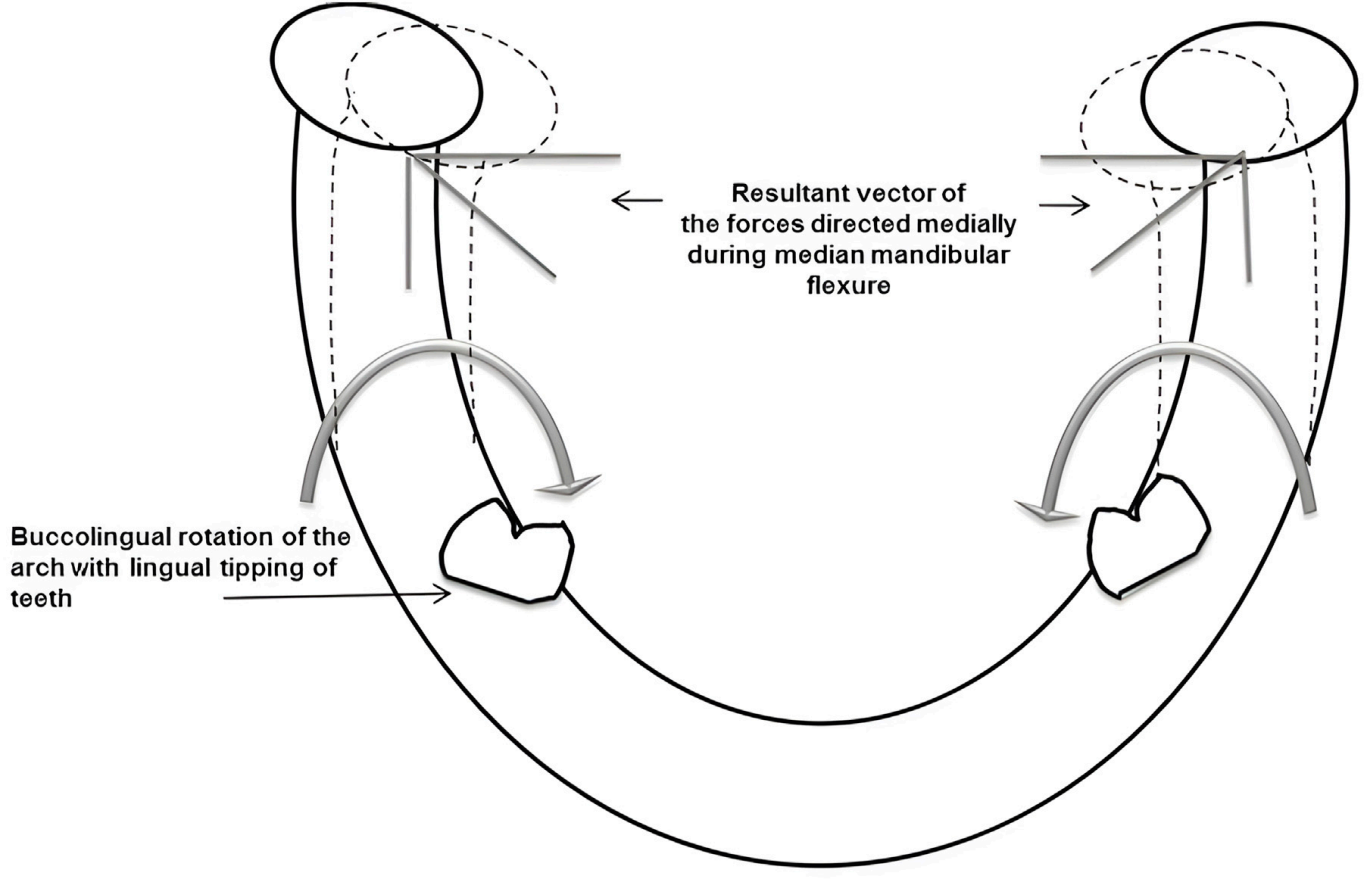
Bucco-lingual tipping of the teeth in lower arch and stress concentration at mandibular symphysis area during mandibular flexure (Sivaraman et al., 2016).

Among the various mandibular deformations, the median mandibular flexure (MMF) is the most influential mandibular deformation mode on implant restoration. Most researchers have reached an initial consensus on the mechanism of mandibular deformation: the “U”-shaped or horseshoe-shaped mandible functions as a curvilinear beam that supports bilateral and unilateral loads. The lateral pterygoid muscles contract to initiate mandibular movement. In conjunction with the sagittal movement of the posterior segment, the medial pull on the mandibular condyle facilitates mandibular flexion around the mandibular symphysis. As depicted in Figure 2, the tension exerted by these attached structures induces changes in the mandibular shape, resulting in a narrower arch and affecting the teeth relative positioning within the mandibular arch (Sivaraman et al., 2016). Therefore, functional flexure of the mandible is worthy of significant biomechanical consideration. Torsional stresses may develop within the mandibular dental arch due to the rigid splinting of natural teeth or the integration of implants via fixed trans-arch bridges. Due to the adaptable nature of the periodontal ligament, these stresses can be compensated for in the case of natural teeth. Significantly, such stresses may also manifest in the superstructure of restorations, potentially leading to ceramic fractures and adhesive failures (Fischman, 1990).

Clinicians must be aware of mandibular deformation, and while there have been notable advances in MF-related research, this area remains relatively understudied, primarily due to technical and methodological limitations. Thus, the authors comprehensively reviewed the literature from 1955 to 2023, including review papers, clinical trials, biomechanical experiments, and case reports. This review aimed to thoroughly explore the mechanisms of MMF and its clinical significance and shed light on the preventative measures clinicians should consider. Moreover, this review endeavored to present novel research ideas and avenues for advancing our understanding of MMF.

## 2 MMF

### 2.1 Deformation caused by MMF

The jaw deforms in at least three directions due to non-masticatory physiological movements, with a deformation range of a few microns to 1 mm and an average value of approximately 0.073 mm (Shinkai et al., 2004; Covani et al., 2011). Furthermore, approximately 2% of patients can experience more than 4 mm elastic displacement of the mandibular condyles during mandibular movement (Linkow and Ghalili, 1999).

During jaw opening and protrusion, the bilateral pterygoid muscles can narrow the mandibular arch by contraction (Manzi et al., 2013). In their study using oral impressions with varying degrees of mandibular opening, Regli CP et al. found that deformation had a positive correlation with opening degree and extension distance (Regli and Kelly, 1967). In addition, Ioanid N demonstrated no change in the width of the mandible up to a mouth opening of 28%, after which the change in width was directly proportional to the mouth opening (Ioanid et al., 2017). Using intraoral scanning, Gülsoy M. demonstrated that MMF values increased linearly from the anterior to the posterior mandible in both dentulous and edentulous individuals (Gülsoy et al., 2022). The posterior portion of the mandibular foramen is more susceptible to elastic deformation, whereas the region between the bilateral mental foramen is relatively stable and less prone to deformation (English, 1993). These findings align with Carl E. Mischs observations, indicating that the cohesive mobility of the first molar can reach 0.8 mm, while that of the mandibular ascending ramus can reach 1.5 mm (Carl, 2005).

In addition to opening and protrusion, flexural movement occurs during lateral and retrusion movements. Hobkirk JA et al. measured relative motion and force transmission between dental osseointegrated implants in the edentulous mandibular premolar region using sensors connected to the implants. It was observed that forces during lateral deflections were significantly less than during open-mouth and closed-mouth movements (Hobkirk and Schwab, 1991), with an average of approximately 0.243–0.257 mm (ElSyad et al., 2019). Moreover, centric relation position (C.R.) is also associated with some amount of mandibular flexure. The study by Omar and Wise measured mandibular flexure in the horizontal plane, recorded using an “anterior jig”, chin-point guidance and patient-exerted muscle forces. The study found that horizontal mandibular retraction forces in the centric relation recordings resulted in an increase in the width of the dental arch (Omar and Wise, 1981; Ioanid et al., 2017).

### 2.2 MMF concurrent deformation types

(Law et al., 2012) reported discovering three deformation patterns in the MMF: symphyseal bending, corporal rotation, and dorsoventral shear. (Abdel-Latif et al., 2000). adopted a displacement sensor to measure the displacement of the most distal implants in patients with the implant-supported fixed denture in the edentulous mandible during lateral, opening, and clenching movements. The distance between the most distal implants on the left and right sides was the variable of convergence between the condyles. Meanwhile, the relative rotation of the left and right mandibular bodies projected on the median sagittal plane was used as the dorsoventral shear, and the rotation of the most distal implant was taken as the corporals degree of rotation. They discovered that the cohesive deformation caused by MMF was 0.04 mm, the degree of body rotation was 60°, and the dorsal-ventral incision was 19°. In addition, (Sesma et al., 1996), placed a displacement sensor close to the implants midline to measure the mandible deformation caused by MMF. Table 1 demonstrates the results.

**TABLE 1:** Deformation of the most distal implants in edentulous patients during mandibular movement (Sesma et al., 1996).

Mandibular movement	Deformation types		
	Condyles convergence (mm)	Corporal rotation (°)	Dorsoventral shear (mm)
Jaw opening movement	0.1–0.04	0.05–0.11	0.04–0.09
Mandibular protrusion movement	0.01–0.02	0.03–0.08	0.03–0.05
Mandibular lateral movement	0.02–0.05	0.03–0.15	0.05–0.1

### 2.3 Factors influencing MMF

#### 2.3.1 Gender

In forensic medicine, the accuracy of mandibular ramus flexure for gender judgment can range from 50% to 80% (Hazari et al., 2016). Remarkably, the Korean populations MF upper border (MFUB) has the highest accuracy in gender discrimination analysis. It has been used in forensic science and law to determine the gender of the Korean population (Lin et al., 2014). Experimentally, Johnson RB et al. observed gender differences in MF, with females generally exhibiting greater flexure than males. This disparity could be attributed to variations in bone density, hormonal influences, and masticatory musculature (Johnson et al., 2009). However, numerous researchers have stated that there is no significant correlation between gender and the deformation of MMF and that the influence of gender on MF may be negligible when compared to other factors, such as occlusal force magnitude, mandibular morphology, and dental occlusion (Wolf et al., 2019; Gülsoy et al., 2022). Also demonstrated that the difference is not statistically significant despite the MMF deformation degree observed in female subjects being greater than in male subjects (Gülsoy et al., 2022). Hence, the possible link between MF and gender remains a topic of ongoing research and discussion.

#### 2.3.2 Age

Age plays a pivotal role in MF, as changes in mandibular bone composition and structure occur with age. Numerous studies have shown that aging is associated with many typical cell-intrinsic factors within the skeleton (Rebelo-Marques et al., 2018) and intrinsic changes in osteolymphatic endothelial cells leading to their lack of stress response to genotoxic agents in the aging skeleton (Biswas et al., 2023); age-dependent perturbation of the vascular niche can also affect skeletal and hematopoietic regeneration in aging animals (Chen et al., 2021). Due to decreased bone mineral density and changes in bone architecture, MF is frequently increased in the elderly (Hobkirk and Schwab, 1991). According to experimental studies, advanced age is associated with elevated levels of MF during functional movements, which impacts the stability and longevity of restorations and implants (Smith et al., 2005).

#### 2.3.3 Bone density

Mandibular bone density is a critical factor influencing the extent of MF. Studies were conducted by (Hylander, 1984; White et al., 2016) to investigate the effect of bone density on MF during functional movements. A strain gauge was employed to measure the extent of MF during functional movement in both experiments involving a cohort of patients with varying bone density. The existence of an inverse correlation between bone density and MF was confirmed by the experiments. Individuals with greater bone density typically exhibit diminished flexure, as denser bones can better withstand mechanical stress during functional movements (Hylander, 1984).

#### 2.3.4 Musculature strength

The ability of the mandible to flex under load is directly linked to the muscle forces exerted on it (Koolstra and van Eijden, 2005), and the direction and intensity of the muscle forces influence the pattern of MF during various functional activities (Lobbezoo et al., 2004). Strong masticatory muscles can generate greater chewing forces. As the muscles contract with greater force, the mandibles bending moment increases, leading to greater flexure (Rafferty et al., 2008). In addition, Green SE et al. examined the impact of muscle imbalances on MF. They found that weaker or unbalanced musculature could increase MF and potential complications in implant-supported prostheses (Green et al., 2020).

According to the positive correlation between masticatory muscle strength and occlusal force (van der Bilt et al., 2006; Ebadian et al., 2020) evaluated the relationship between mandibular occlusal force (MOF) and MMF in a cohort of adult participants, revealing that MOF and MMF are critical and effective factors in the success of prosthetic restorations. Nonetheless, (Canabarro Sde and Shinkai, 2006), employed a distinct methodology in their study, which collected bilateral MOF measurements using transarch force transducers positioned in the first molar region. The mandibular occlusal surface impressions were obtained at rest (R), maximal opening (O), and maximal inclination (P). The degree of MF was then computed using these impressions. This approach discovered no significant association between MMF and MOF in this group of dentate adults.

#### 2.3.5 Symphyseal bone height

MF deformation is influenced by the height of the symphyseal bone, which represents the region where the two-halves of the mandible meet. (Chen et al., 2000) investigated the relationship between the symphyseal bone height and MF during functional movements. The study found a significant positive association between the symphyseal bone height and decreased MF. Individuals with a greater symphyseal bone height exhibited improved stability of the mandible during functional movements, leading to reduced flexure (Canabarro Sde et al., 2006).

#### 2.3.6 Lower gonial angle

The lower gonial angle, formed between the mandibular ramus and the mandibular body, is another significant factor affecting MF. A smaller angle is associated with greater mandibular flexibility, contributing to higher MF levels (Shinkai et al., 2004). In contrast, a larger angle increases stability and decreases flexure during functional movements (Suresh, 2005).

#### 2.3.7 Facial type

The population can be classified into three main facial types: short-faced, medium-faced, and long-faced. There is a significant correlation between the vertical facial pattern and the thickness of the masseter muscle, resulting in varying masticatory muscle strength among face types. Robust or thick masticatory muscles impose an increased mechanical load on the jaws, stimulating suture growth and bone alignment, ultimately leading to lateral jaw growth (Van Spronsen et al., 1992; Satiroğlu et al., 2005; Prasad et al., 2013) measured experimentally the variation in the distance between bilateral first molars at the maximum open and resting positions for three facial types in the South Indian population. The findings revealed a correlation between facial pattern variation and MMF values, with the short facial type displaying the highest MMF levels, followed by the medium and long facial types. Moreover, (Custodio et al., 2011), study involved measuring MMF values among three distinct facial types, concluding that vertical facial type affected MF. However, there are also contradictory studies. Shinkai RS et al. analyzed the correlation between the three facial types and MMF in a Brazilian population using impression-making and found no relationship between MMF and vertical facial morphology (Shinkai et al., 2007). In conclusion, the correlation between MMF and facial type is still debatable and requires further investigation.

## 3 The impact of MMF on implant restoration

MMF is one of the essential clinical factors affecting the design of dentures and their subsequent clinical effects. Its undesirable consequences include 1) the inability to passively position the prosthesis due to an imprecise impression; 2) screw or superstructure fracture; 3) bone loss surrounding the distal implants; and 4) fatigue and fracture of metal materials due to repeated compression (Mijiritsky et al., 2022). Therefore, a comprehensive understanding of the clinical significance of this biological phenomenon in implant restorations and the precise diagnostic and therapeutic protocols followed are essential for prolonging denture longevity and improving patient satisfaction.

### 3.1 Accuracy of making impressions

In order to create a clinical impression, a certain degree of opening is required. The traditional wide-opening impression technique will cause muscle contraction, resulting in the cohesion of the mandible and the reduction of the dental archs width, which ranges between 0.011 mm and 0.232 mm (Wolf et al., 2019). Furthermore, the teeth will be positioned more lingually than the intercuspal position (ICP). Additionally, the pressure placed on a patients jaw by dentists during an impression can cause changes to the mandibles width. If the mandibular impression is made at the opening stage, the restoration made on the corresponding working model can only achieve a proper fit at the opening stage. The mismatches will induce significant differences between the final restoration and the patients oral condition, preventing the passive fit of the final fixed and removable dentures. When the denture is worn to perform functions, the teeth and restorations are susceptible to undesirable stress, causing occlusal interference, pain and discomfort, bone resorption, gingivitis, and other complications.

Therefore, when making impressions, controlling the patients extent of opening is crucial. Using the closed-mouth technique to make an impression can minimize the contraction of the masticatory muscles and reduce mandibular deformation. When making an impression, (Gates and Nicholls, 1981), believed that applying a horizontal retraction force to the mandible could prevent width reduction. (Omar and Wise, 1981). suggested that any opening and protrusive movements exceeding 20 mm should be avoided when making impressions to minimize the amount of change in the width of the mandibular arch.

### 3.2 Recording centric relation

During patient-guided Centric Relation (C.R.) registration or functional procedures, MF may impact the restorations fit, leading to challenges in achieving proper occlusal contact (Sivaraman et al., 2016). The horizontal plane MF can cause a discrepancy between the cusp indentation in the jaw registration record and the cusp position on the dental cast (Kan et al., 1999). Due to the lingual movement of the mandibular teeth, the occlusal relationship may be inaccurately represented, and prostheses fabricated from such records may exhibit occlusal interference. The results of Omar and Wise’s study showed greater mandibular flexure when centric relationships are recorded by patient-guided muscular movements (Omar and Wise, 1981). To minimize this discrepancy, it is advisable to utilize the “closed mouth” impression technique and the C.R. technique, as the dentist directs, for recording. Additionally, the closed-mouth occlusal double arch method, supported by (Tylman, 1989), can help in avoiding the MF effect associated with the open-mouth technique.

### 3.3 Implant supported overdenture

MF, which can impact the fit of removable implant-supported overdentures, should be considered during fabrication. For the fabrication of implant frameworks, computer-aided design/computer-aided manufacturing (CAD/CAM) is a superior option. However, it is also susceptible to MF (Kan et al., 1999; Torsello et al., 2008) due to its large camera head, which may necessitate a wide lower jaw opening for insertion. Future mitigation of the impact of MF on the CAD/CAM technique may be possible due to ongoing progress in this aspect (Sivaraman et al., 2016).

### 3.4 The segmented design of the fixed implant-supported superstructure

In the context of fixed prostheses, MF can pose particular challenges. The segmented design of the superstructure for implant-supported fixed dentures in edentulous patients is currently debated. (Martin-Fernandez et al., 2018). experiments revealed that a one-piece superstructure provides the optimal biomechanical environment for mandibular movement. In contrast, the two-piece and three-piece models exert greater stress on restoration components during protrusion and opening, respectively. Moreover, a study utilized three-dimensional finite element analysis to investigate the impact of MF on the segmental design of fixed frameworks for edentulous implants. According to the study, the greatest stress in one-piece framework restorations occurred around the distal implants on both sides, progressively decreasing toward more mesial positions. In two-piece framework restorations, the greatest stress was observed surrounding the lateral incisor location. The three-piece framework restorations exhibited greater stress than the one-piece and two-piece frameworks, where the maximum stress was observed around the canine location. Thus, patients with edentulous implant-supported fixed restorations may benefit from a non-segmented framework without a cantilever, as it provides an optimal biomechanical environment (Gao et al., 2022). These findings support the theory that an inflexible full-arch prosthesis can provide additional resistance, thereby offsetting the effects of MF, particularly in cases with a single unilateral posterior framework (Naini and Nokar, 2009; Zaugg et al., 2012).

However, more researchers prefer a segmented superstructure design for edentulous patients. Zarone F et al. suggested that the flexibility of the implant-restored mandible is affected by at least two factors: the position of the implants and the type of prosthetic superstructure. As a result of mandibular functional flexure in mandibular full-arch fixed prostheses supported by osseointegrated implants, a substantial amount of stress develops in the more distal implants and the superstructure at the symphysis (Zarone et al., 2003). In this way, the segmented framework can better adapt to the physiologic curvature characteristics of the mandible, guarantee accurate and passive implant placement, and increase the restorations service life (Suedam et al., 2009; Marin et al., 2015; Azpiazu-Flores et al., 2022). When using a cross-arch implant-supported fixed denture to restore edentulous jaws, failure to consider MMF can lead to mechanical and biological complications, resulting in pain and discomfort for the patient. With more implants connected by rigid splints and a longer dental arch, the risk of MMF negatively affecting implant or restoration prognosis increases. (Marin et al., 2015). demonstrated that dividing the superstructure into two parts at the symphyseal region only reduces stress caused by MMF, whereas dividing it into three parts is more effective in mitigating the effect of mandibular rotation and achieving superior clinical outcomes. Other clinical trials (Paez et al., 2003) demonstrated that separating the superstructure from the midline reduces patient pain and discomfort, further improving when the superstructure is divided into three parts. Furthermore, (Nokar and Baghai Naini, 2010), confirmed that stress on each restoration component and the jaw differs between two-segment and three-segment superstructures when occlusal forces are applied at different positions (two static bites of the incisal and right molar clenching). Table 2 summarizes the findings. Consequently, the authors propose that a two-piece superstructure provides a more favorable biomechanical environment for molar occlusion, whereas a three-piece superstructure is more suited for incisor occlusion.

**TABLE 2:** When the bite force is loaded at different positions (two static bites of incisal and right molar clenching), the maximum stress point and deformation of the two-piece and three-piece superstructure (Nokar and Baghai Naini, 2010).

Clenching	Superstructure design	Maximum stress values (MPa)	Deformation (mm)
Incisal clenching (INC)	Two- piece superstructure	Condylar region, masseter attachment, buccal cortical bone of the most mesial implant (29.1)	Mandibular angle (0.6) Symphyseal region (0.45)
	Three- piece superstructure	Buccal cortical bone of the most mesial implant (20.1)	Mandibular angle (0.55) Symphyseal region (0.3)
Right molar clenching (RMOL)	Two- piece superstructure	Mandibular ramus, condyle, the lingual aspect of cortical bone around the most distal implant on the right side (104.7)	Mandibular angle and lower edge of Symphyseal region (0.3)
	Three-piece superstructure	Lingual aspect of cortical bone around the most distal implant on the right side (62.5)	Mandibular angle (0.5) Symphyseal region (0.6)

The segmental design of the superstructure remains controversial in light of these findings. In addition, it is essential to tailor the restorations design to the patients occlusal habits, including group functional occlusion, canine protection, and balanced occlusion, to maximize the restorations longevity. Therefore, additional exhaustive and in-depth research is required to confirm the results.

### 3.5 Bone resorption

Studies have shown that MMF is a major factor in implant and superstructure loosening in implant-supported fixed dentures (Sivaraman et al., 2016). This phenomenon is caused primarily by the differences between natural teeth and dental implants (Azpiazu-Flores et al., 2022). Through the periodontal ligament, natural teeth have a close integration with the alveolar bone, which allows for the adjustment and buffering of bite forces and provides sensory feedback to protect the alveolar bone. During functional movement, teeth exhibit a degree of physiological activity (approximately 28 µm vertically (Borg and Grondahl, 1996) and 56–75 µm horizontally (Wenzel and Gröndahl, 1995)). Dental implants, on the other hand, integrate directly with the alveolar bone via osseointegration (Varthis et al., 2019), resulting in limited mobility (with a maximum vertical and horizontal range of only 2–3 µm) (Borg and Grondahl, 1996). These differences decrease peri-implant tissue adaptability and tolerance compared to natural teeth.

Due to micromovements, MMF may cause microdamage at the crestal region and suboptimal osseointegration around implants. During mandibular movement, MMF flexes and twists with the median joint as the pivot; considerable stress is generated in the neck of the implant and the midline of the superstructure (Hobkirk and Havthoulas, 1998), leading to bone compression and subsequent loss (Zarone et al., 2003; Hobkirk and Schwab, 1991) discovered posterior implants in cantilever situations may experience stress-induced microdamage at the bone-implant interface due to MF. Lindquist et al., using stereoscopic intra-oral radiography, discovered pronounced crestal bone loss around anterior implants compared to posterior implants in the symphysis region. It was primarily due to the restricting effect of the splint at the primary point of flexure (Lindquist et al., 1988). Moreover, it has been observed that using fewer implants can result in localized force distribution patterns (Marin et al., 2015). To address this, researchers advocate increasing the number of implants in edentulous jaws, with feasible placement in the posterior regions based on anatomical and surgical considerations, to minimize cantilever situations and excessive spacing between implants (Gao et al., 2022). Fixed implants and flexible connectors are discouraged in the anterior region, and two or more independent restorations are preferred (Borg and Grondahl, 1996). It is recommended to use freestanding mandibular posterior osseointegrated implants with fixed restorations featuring shorter spans or stress relievers attached to the abutment to optimize the treatment outcome when implant size or bone quality is problematic.

### 3.6 Selection of superstructure materials

MMF plays a crucial role in selecting materials for implant denture superstructures. Implant-supported rigid splints can limit the extent of MF, and the degree of inhibition is proportional to the number of connected implants and the materials stiffness (Favot et al., 2014; Abadzhiev et al., 2017) reported that MMF contributes to chronic pain syndrome in edentulous patients with implant-supported fixed restorations. (Favot et al., 2014). conducted three-dimensional finite element analysis to compare mandibular deformations in edentulous patients with restored occlusion using four different materials (zirconia, titanium, gold, and nickel-titanium (NiTi)). The results indicated that mandibular deformation decreases with increasing material stiffness, with NiTi exhibiting the best adaptation to MF throughout all chewing stages, followed by gold, titanium, and zirconium oxide. Matching the elastic modulus of the superstructure material to that of bone tissue or employing a material with a high elastic modulus comparable to that of bone tissue is crucial for avoiding stress on the bone tissue around the distal implant and minimizing potential complications such as discomfort, pain, and bone resorption caused by excessive stress.

## 4 Conclusion

Clinicians must implement appropriate preventive measures and precise clinical techniques to minimize mandibular flexure during diagnosis and treatment of implant restorations. A careful regulation of the degree of a patients mouth opening during impression-taking and consideration of a superstructure material with a high elastic modulus comparable to the elastic modulus of bone tissue can contribute to the achievement of optimal restorations while preserving the health of periodontal tissue and bone tissue. However, there are ongoing discussions regarding the choice of segmental design for the superstructure of implant-supported fixed dentures in edentulous patients. Exploring adjustments to implant denture design based on different facial types and occlusal habits can yield valuable insights and references that can be used to optimize clinical diagnosis and treatment.

## Abbreviations

MF, mandibular flexure; MMF, median mandibular flexure; MOF, maximum occlusal force; MFUB, mandibular flexure upper border; ICP, intercuspal position; NiTi C.R., nickel-titanium Centric Relation.

## References

[B1] Abadzhiev M. Todorov G. Kamberov K. (2017). Mandibular flexure – a REASON FOR CHRONIC PAIN SYNDROME IN EDENTULOUS PATIENT RESTORED WITH FIXED ZRO2 CONSTRUCTION OVER IMPLANTS, INSERTED IN NATURAL BONE AND BONE GRAFT AREA. Case report. Annu. Proceeding Sci. Pap. 23, 1432–1440. 10.5272/jimab.2017231.1432

[B2] Abdel-Latif H. H. Hobkirk J. A. Kelleway J. P. (2000). Functional mandibular deformation in edentulous subjects treated with dental implants. Int. J. Prosthodont 13, 513–519. 11203678

[B3] Azpiazu-Flores F. X. Mata-Mata S. J. Lee D. J. (2022). Detection of mandibular flexure with a dental plaster verification device: A clinical report with video recording. J. Prosthet. Dent. S0022-3913 (22), 105–106. 10.1016/j.prosdent.2022.01.039 35465960

[B4] BiswasL.ChenJ.De AngelisJ.SinghA.Owen-WoodsC.DingZ. (2023). Lymphatic vessels in bone support regeneration after injury. Cell 186 (2), 382–397. 10.1016/j.cell.2022.12.031 36669473

[B5] Borg E. Grondahl H. G. (1996). On the dynamic range of different x-ray photon detectors in intra-oral radiography. A comparison of image quality in film, charge-coupled device and storage phosphor systems. Dentomaxillofac Radiol. 25, 82–88. 10.1259/dmfr.25.2.9446978 9446978

[B6] Canabarro Sde A. Shinkai R. S. (2006). Medial mandibular flexure and maximum occlusal force in dentate adults. Int. J. Prosthodont 19 (2), 177–182. 16602367

[B7] Carl E. (2005). “Misch. The completely edentulous mandible: treatment plans for fixed restorations,” in Misch C.E. Dental implant prosthetics (St. Louis: Elsevier Mosby), 602.

[B8] Chen D. C. Lai Y. L. Chi L. Y. Lee S. Y. (2000). Contributing factors of mandibular deformation during mouth opening. J. Dent. 28, 583–588. 11082527 10.1016/s0300-5712(00)00041-5

[B9] Chen J. Sivan U. Tan S. L. Lippo L. De Angelis J. Labella R. (2021). High-resolution 3D imaging uncovers organ-specific vascular control of tissue aging. Sci. Adv. 7 (6), eabd7819. 10.1126/sciadv.abd7819 33536212 PMC7857692

[B10] Covani U. Ricci M. Bozzolo G. Mangano F. Zini A. Barone A. (2011). Analysis of the pattern of the alveolar ridge remodelling following single tooth extraction. Clin. Oral Implants Res. 22 (8), 820–825. 10.1111/j.1600-0501.2010.02060.x 21198897

[B11] Custodio W. Gomes S. G. Faot F. Garcia R. C. Del Bel Cury A. A. (2011). Occlusal force, electromyographic activity of masticatory muscles and mandibular flexure of subjects with different facial types. J. Appl. Oral Sci. 19 (4), 343–349. 10.1590/s1678-77572011005000008 21655772 PMC4223785

[B12] Ebadian B. Abolhasani M. Heidarpour A. Ziaei M. Jowkar M. (2020). Assessment of the relationship between maximum occlusal force and median mandibular flexure in adults: A clinical trial study. J. Indian Prosthodont Soc. 20 (1), 76–82. 10.4103/jips.jips_282_19 32089602 PMC7008623

[B13] ElSyad M. A. Alameldeen H. E. Elsaih E. A. (2019). Four-implant-supported fixed prosthesis and milled bar overdentures for rehabilitation of the edentulous mandible: A 1-year randomized controlled clinical and radiographic study. Int. J. Oral Maxillofac. Implants 34, 1493–1503. 10.11607/jomi.7667 31184639

[B14] English C. E. (1993). Biomechanical concerns with fixed partial dentures involving implants. Implant Dent. 2, 221–241. 10.1097/00008505-199312000-00002 8004049

[B15] Favot L. M. Berry-Kromer V. Haboussi M. Thiebaud F. Ben Zineb T. (2014). Numerical study of the influence of material parameters on the mechanical behaviour of a rehabilitated edentulous mandible. J. Dent. 42 (3), 287–297. 10.1016/j.jdent.2013.11.027 24321295

[B16] Fischman B. (1990). The rotational aspect of mandibular flexure. J. Prosthet. Dent. 64, 483–485. 10.1016/0022-3913(90)90049-i 2231461

[B17] Gao J. Li X. He J. Jiang L. Zhao B. (2022). The effect of mandibular flexure on the design of implant-supported fixed restorations of different facial types under two loading conditions by three-dimensional finite element analysis. Front. Bioeng. Biotechnol. 10, 928656. 10.3389/fbioe.2022.928656 36105608 PMC9465293

[B18] Gates G. N. Nicholls J. I. (1981). Evaluation of mandibular arch width change. J. Prosthet. Dent. 46, 385–392. 10.1016/0022-3913(81)90443-1 6946212

[B19] Goodkind R. J. Heringlake C. B. (1973). Mandibular flexure in opening and closing movements. J. Pros. Dent. 30, 134–138. 10.1016/0022-3913(73)90046-2 4515668

[B20] Green S. E. Johnson A. B. Williams E. F. Davis G. H. (2020). Impact of muscle imbalances on mandibular flexure. J. Oral Rehabil. 47 (9), 1081–1087.

[B21] Gülsoy M. Tuna S. H. Pekkan G. (2022). Evaluation of median mandibular flexure values in dentulous and edentulous subjects by using an intraoral digital scanner. J. Adv. Prosthodont 14 (1), 32–44. 10.4047/jap.2022.14.1.32 35284055 PMC8891685

[B22] Hazari P. Hazari R. S. Mishra S. K. Agrawal S. Yadav M. (2016). Is there enough evidence so that mandible can be used as a tool for sex dimorphism? A systematic review. J. Forensic Dent. Sci. 8 (3), 174. 10.4103/0975-1475.195111 28123276 PMC5210109

[B23] Hobkirk J. A. Havthoulas T. K. (1998). The influence of mandibular deformation, implant numbers, and loading position on detected forces in abutments supporting fixed implant superstructures. J. Prosthet. Dent. 80, 169–174. 10.1016/s0022-3913(98)70106-4 9710818

[B24] Hobkirk J. A. Schwab J. (1991). Mandibular deformation in subjects with osseointegrated implants. Int. J. Oral Maxillofac. Implants 6 (3), 319–328. 1813399

[B25] Hylander W. L. (1984). Stress and strain in the mandibular symphysis of primates: A test of competing hypotheses. Am. J. Phys. Anthropol. 64, 1–46. 10.1002/ajpa.1330640102 6731608

[B26] Ioanid N. Țănculescu O. Luca O. MârţuStefanache M. A. Doscas A. R. Ciofu M. (2017). Study on mandibular medial flexure value (MMF) for natual tooth and dental implant support. Ro J. Oral Rehabil. 9, 99–108.

[B27] Johnson R. B. White J. S. Andersson E. M. (2009). Gender differences in mandibular flexure. J. Prosthet. Dent. 101 (5), 307–314.

[B28] Kan J. Y. Rungcharassaeng K. Bohsali K. Goodacre C. J. Lang B. R. (1999). Clinical methods for evaluating implant framework fit. J. Prosthet. Dent. 81 (1), 7–13. 10.1016/s0022-3913(99)70229-5 9878969

[B29] Koolstra J. H. van Eijden T. M. (2005). Combined finite-element and rigid-body analysis of human jaw joint dynamics. J. Dent. Res. 84 (1), 2431–2439. 10.1016/j.jbiomech.2004.10.014 16214491

[B30] Law C. Bennani V. Lyons K. Swain M. (2012). Mandibular flexure and its significance on implant fixed prostheses: A review. J. Prosthodont 21 (3), 219–224. 10.1111/j.1532-849X.2011.00798.x 22044758

[B31] Lin C. Jiao B. Liu S. Guan F. Chung N. E. Han S. H. (2014). Sex determination from the mandibular ramus flexure of Koreans by discrimination function analysis using three-dimensional mandible models. Forensic Sci. Int. 236, 191.e1–6. 10.1016/j.forsciint.2013.12.015 24439155

[B32] Lindquist L. Rockler B. Carlsson G. (1988). Bone resorption around fixtures in edentulous patients treated with mandibular fixed tissue integrated prostheses. J. Prosthet. Dent. 59, 59–63. 10.1016/0022-3913(88)90109-6 3422305

[B33] Linkow L. I. Ghalili R. (1999). Ramus hinges for excessive movements of the condyles: A new dimension in mandibular tripodal subperiosteal implants. J. Oral Implantol. 25, 11–17. 10.1563/1548-1336(1999)025<0011:RHFEMO>2.3.CO;2 10483422

[B34] Lobbezoo F. Van der Glas H. W. Van Kampen F. M. Bosman F. van der Bilt A. (2004). The effects of jaw clenching and jaw relaxation on the human masseter muscle. Arch. Oral Biol. 49 (4), 281–286.

[B35] Manzi M. R. Manzano R. Pimentel A. C. (2013). Medial mandibular flexure related to biomechanical failures of implant-supported fixed prosthesis with rigid connection distal to the mental foramen. Dent. Press Implantol. 7, 43–50.

[B36] Marin D. O. Dias Kde C. Paleari A. G. Pero A. C. Arioli Filho J. N. Compagnoni M. A. (2015). Split-framework in mandibular implant-supported prosthesis. Case Rep. Dent. 2015, 1–5. 10.1155/2015/502394 PMC4681794 26770841

[B37] Martin-Fernandez E. Gonzalez-Gonzalez I. deLlanos-Lanchares H. Mauvezin-Quevedo M. A. Brizuela-Velasco A. Alvarez-Arenal A. (2018). Mandibular flexure and peri-implant bone stress distribution on an implant-supported fixed full-arch mandibular prosthesis: 3D finite element analysis. Biomed. Res. Int. 2018, 1–9. 10.1155/2018/8241313 PMC5899843 29805978

[B38] Mijiritsky E. Shacham M. Meilik Y. Dekel-Steinkeller M. (2022). Clinical influence of mandibular flexure on oral rehabilitation: narrative review. Int. J. Environ. Res. Public Health 19 (24), 16748. 10.3390/ijerph192416748 36554629 PMC9778818

[B39] Naini R. B. Nokar S. (2009). Three-dimensional finite element analysis of the effect of 1-piece superstructure on mandibular flexure. Implant Dent. 18 (5), 428–437. 10.1097/ID.0b013e3181ad8d87 22129961

[B40] Nokar S. Baghai Naini R. (2010). The effect of superstructure design on stress distribution in peri-implant bone during mandibular flexure. Int. J. Oral Maxillofac. Implants 25 (1), 31–37. 20209184

[B41] Omar R. Wise M. D. (1981). Mandibular flexure associated with muscle force applied in the retruded axis position. J. Oral Rehabil. 8, 209–221. 10.1111/j.1365-2842.1981.tb00495.x 6942134

[B42] Paez C. Y. Barco T. Roushdy S. Andres C. (2003). Split-frame implant prosthesis designed to compensate for mandibular flexure: A clinical report. J. Prosthet. Dent. 89 (4), 341–343. 10.1067/mpr.2003.57 12690344

[B44] Prasad M. Hussain M. Z. Shetty S. K. Kumar T. Khaur M. George S. (2013). Median mandibular flexure at different mouth opening and its relation to different facial types: A prospective clinical study. J. Nat. Sci. Biol. Med. 4, 426–430. 10.4103/0976-9668.117028 24082745 PMC3783793

[B45] Rafferty K. L. Liu Z. J. Ye W. Major P. W. (2008). Head posture and the morphology of the first cervical vertebra (Atlas). Spine 33 (1), 76–82.

[B46] Rebelo-Marques A. De Sousa Lages A. Andrade R. Ribeiro C. F. Mota-Pinto A. Carrilho F. (2018). Aging hallmarks: the benefits of physical exercise. Front. Endocrinol. (Lausanne). 9, 258. 10.3389/fendo.2018.00258 29887832 PMC5980968

[B47] Regli C. P. Kelly E. K. (1967). The phenomenon of decreased mandibular arch width in opening movements. J. Prosthet. Dent. 17 (1), 49–53. 10.1016/0022-3913(67)90050-9 5224788

[B48] Satiroğlu F. Arun T. Işik F. (2005). Comparative data on facial morphology and muscle thickness using ultrasonography. Eur. J. Orthod. 27, 562–567. 10.1093/ejo/cji052 16135538

[B49] Sesma N. Ribeiro F. C. Zanetti A. L. (1996). Delexão mandibular e suas relações com próteses e implantes osseointegrados. Rev. Assoc. Paul. Cir. Dent. 50, 73–77.

[B50] Shinkai R. S. Canabarro Sde A. Schmidt C. B. Sartori E. A. (2004). Reliability of a digital image method for measuring medial mandibular flexure in dentate subjects. J. Appl. Oral Sci. 12 (4), 358–362. 10.1590/s1678-77572004000400020 20976412

[B51] Shinkai R. S. Lazzari F. L. Canabarro S. A. Gomes M. Grossi M. L. Hirakata L. M. (2007). Maximum occlusal force and medial mandibular flexure in relation to vertical facial pattern: A cross-sectional study. Head. Face Med. 3, 18. 10.1186/1746-160X-3-18 17407566 PMC1851008

[B52] Sivaraman K. Chopra A. Venkatesh S. B. (2016). Clinical importance of median mandibular flexure in oral rehabilitation: A review. J. Oral Rehabil. 43, 215–225. 10.1111/joor.12361 26498998

[B54] Smith J. M. Johnson R. B. Anderson E. M. (2005). Age-related changes in mandibular flexure determined using implant strain gauge analysis. J. Oral Rehabil. 32 (2), 117–122.

[B55] Suedam V. Souza E. A. Moura M. S. Jacques L. B. Rubo J. H. (2009). Effect of abutment's height and framework alloy on the load distribution of mandibular cantilevered implant-supported prosthesis. Clin. Oral Implants Res. 20 (2), 196–200. 10.1111/j.1600-0501.2008.01609.x 19191796

[B56] Suresh S. H. (2005). An analysis of mandibular flexure on mouth opening for dentate subjects: in vivo study. dissertation. Chennai: The Tamilnadu: Dr. M.G.R. Medical University.

[B57] Torsello F. Di Torresanto V. M. Ercoli C. Cordaro L. (2008). Evaluation of the marginal precision of one-piece complete arch titanium frameworks fabricated using five different methods for implant-supported restorations. Clin. Oral Implants Res. 19, 772–779. 10.1111/j.1600-0501.2008.01555.x 18720557

[B58] Tylman G. L. (1989). Tylman's theory and practice of fixed prosthodontics. St. Louis: Ishiyaku Euro America, 407–417.

[B59] van der Bilt A. van Kampen F. M. Cune M. S. Bosman F. (2006). Relationship between maximal bite force and facial morphology in young adults. J. Dent. Res. 85 (3), 243–246.

[B60] Van Spronsen P. H. Weijs W. A. Prahl-Andersen Valk J. B. Van Ginkel F. C. (1992). A comparison of jaw muscle cross-sections of long-face and normal adults. J. Dent. Res. 71, 1279–1285. 10.1177/00220345920710060301 1613176

[B61] Varthis S. Tarnow D. P. Randi A. (2019). Interproximal open contacts between implant restorations and adjacent teeth. prevalence-causes-possible solutions. J. Prosthodont 28, 806–810. 10.1111/jopr.12980 30350332

[B62] Weinmann J. P. Sicher H. (1955). Bone and bones: fundamentals of bone biology. Am. J. Med. Sci. 42, 394–395. 10.1097/00007611-194708000-00022

[B63] Wenzel A. Gröndahl H. G. (1995). Direct digital radiography in the dental office. Int. Dent. J. 45 (1), 27–34. 7607741

[B64] White J. S. Johnson A. B. Williams E. F. Davis G. H. (2016). Relationship between bone density and mandibular flexure. J. Dent. Res. 95 (3), 345–350.

[B66] Wolf L. Bergauer B. Adler W. Wichmann M. Matta R. E. (2019). Three-dimensional evaluation of mandibular deformation during mouth opening. Int. J. Comput. Dent. 22 (1), 21–27. 30848251

[B67] Zarone F. Apicella A. Nicolais L. Aversa R. Sorrentino R. (2003). Mandibular flexure and stress build-up in mandibular full-arch fixed prostheses supported by osseointegrated implants. Clin. Oral Implants Res. 14 (1), 103–114. 10.1034/j.1600-0501.2003.140114.x 12562372

[B68] Zaugg B. Hämmerle C. H. Palla S. Gallo L. M. (2012). Implant-supported mandibular splinting affects temporomandibular joint biomechanics. Clin. Oral Implants Res. 23, 897–901. 10.1111/j.1600-0501.2011.02241.x 21689164

